# Sarcopenia in type 2 diabetes mellitus: an imaging review

**DOI:** 10.3389/fmed.2026.1637499

**Published:** 2026-01-21

**Authors:** Lei He, Guanghua Luo, Hongtao Jiang, Li Zhang, Yusheng Li, Wenbin Gu, Qiqun Zeng, Jingjun Zhu, Jincai Liu, Hao Lei, Heng Zhao

**Affiliations:** 1 Department of Radiology, The First Affiliated Hospital, Hengyang Medical School, University of South China, Hengyang, Hunan, China; 2 Department of Radiology, The First Affiliated Hospital, Shaoyang University, Shaoyang, Hunan, China

**Keywords:** sarcopenia, type 2 diabetes mellitus, dual-energy x-ray absorptiometry, computed tomography, magnetic resonance imaging, ultrasound

## Abstract

Sarcopenia is characterized by an age-related decline in muscle mass, strength, or endurance. It is increasingly prevalent in patients with type 2 diabetes mellitus (T2DM) and is now regarded as a key complication of the disease. Additionally, it has a significant impact on patients’ prognosis. Imaging methods are crucial tools for assessing muscle mass and microchanges. Moreover, they can facilitate the early diagnosis of sarcopenia. Thus, this article reviews the pathological basis and clinical manifestations of sarcopenia in T2DM, the advantages and disadvantages of imaging assessment methods, their specific applications, imaging manifestations, and research progress.

## 1 Introduction

Type 2 diabetes mellitus (T2DM) is a disorder of glucose metabolism resulting from insufficient relative insulin secretion and reduced insulin sensitivity. It is characterized by hyperglycemia and is accompanied by polyuria, polydipsia, weight loss, fatigue, and other symptoms (1–3).

In 2024, the Global Leadership Initiative for Sarcopenia (GLIS) reached a consensus noting sarcopenia as a progressive and potentially reversible skeletal muscle disease characterized by loss of muscle mass and strength (4). Consequently, it is associated with a range of adverse outcomes in the geriatric population. These include reduced physical function, diminished cardiopulmonary performance, and ultimately, loss of capacity and death (5). Moreover, sarcopenia is increasingly prevalent in patients with T2DM (6). Both T2DM and sarcopenia share several common risk factors, such as older age, poor dietary intake, smoking, hormonal imbalance, unhealthy lifestyle habit, and vitamin D deficiency. Sarcopenia has been formally recognized as a diabetes-related complication (7). In T2DM patients, poor metabolic regulation, diabetes duration, and diabetes-related complications exacerbate the occurrence and progression of sarcopenia (8–10). This significantly impacts patients’ quality of life and prognosis, while also increasing the economic burden on families, society, and the healthcare system (11–14).

Dual-Energy X-Ray Absorptiometry (DXA), Computed Tomography (CT), Magnetic Resonance Imaging (MRI), and other imaging methods can reveal the imaging characteristics of sarcopenia in diabetic patients. Importantly, they can reflect the pathological process to some extent and are valuable for early disease recognition, severity assessment, treatment effect evaluation, and prognosis.

## 2 The pathophysiological basis for sarcopenia in T2DM

**Figure 1:**
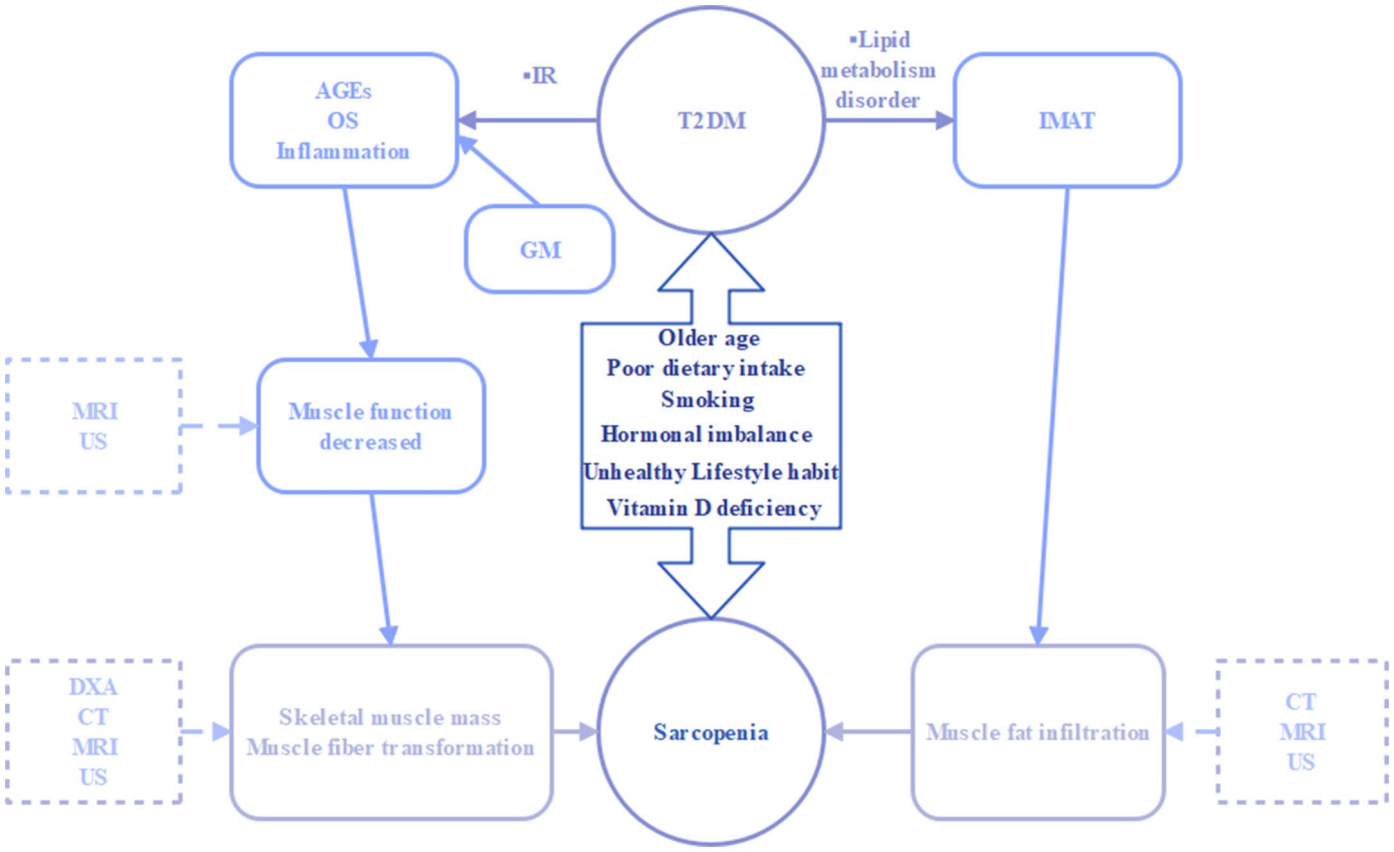
Interaction between type 2 diabetes mellitus and sarcopenia under common risk factors. IR, insulin resistance; AGEs, advanced glycation end-products; OS, oxidative stress; GM, gut microbiota; IMAT, intramuscular adipose tissue; DXA, dual-energy X-ray absorptiometry; CT, computed tomography; MRI, magnetic resonance imaging; US, ultrasound.

The etiology of sarcopenia in diabetic patients is complex and multifactorial. This section examines several key factors that contribute to a deeper understanding of imaging signs. These factors include insulin resistance, oxidative stress, mitochondrial dysfunction, the accumulation of glycation end-products, the presence of intramuscular adipose tissue (IMAT), inflammatory responses, and the composition of the intestinal microbiota (Figure 1).

### 2.1 Insulin resistance (IR)

IR is a key pathogenic mechanism in T2DM. Notably, it accelerates protein breakdown and impairs new protein synthesis, reducing muscle mass and strength (15). On the one hand, IR inhibits protein synthesis by blocking the IGF-1 pathway via the rapamycin target protein mechanism (16). As muscle mass decreases, IR also promotes adipose tissue breakdown, releasing free fatty acids (FFA), this release further inhibits the IGF-1 pathway and protein synthesis (17). On the other hand, IR speeds up protein decomposition by activating the ubiquitin-proteasome system (18). Concurrently, hyperinsulinemia enhances the myostatin-activated p38-caspase signaling pathway that triggers apoptosis, increasing protein decomposition (19–21).

### 2.2 Oxidative stress

Oxidative stress predominantly stems from an escalation in reactive oxygen species (ROS) (22). In individuals with T2DM, the persistent state of hyperglycemia escalates the production and aggregation of ROS by amplifying the substrate pool (23) and activating key enzymes like protein kinase C and NADPH oxidase (24). As a result, elevated ROS levels accelerate muscle protein degradation via the ubiquitin-proteasome system (25), inhibit the Akt/mTOR signaling pathway and downstream protein synthesis (26). Furthermore, heightened oxidative stress damages DNA and impairs satellite cell differentiation (27). In summary, the primary impact of T2DM oxidative stress on sarcopenia is the inhibition of muscle regeneration and an increase in muscle breakdown.

### 2.3 Inflammation

Studies have demonstrated raised circulating C-reactive protein (CRP) and pro-inflammatory cytokines, such as tumor necrosis factor alpha (TNFα) and interleukin 6 (IL-6), in T2DM patients (28–30). In obesity-related T2DM, muscle tissue has upregulated pro-inflammatory pathways such as chemokine (C-C motif) ligand 2 (CCL2), signal transducer and activator of transcription 3 (STAT3), suppressor of cytokine signaling 3 (SOCS3), and nuclear factor kappa B (NF-κB) (31–33). Consequently, these are linked to increased skeletal muscle catabolism (34). As a result, the inflammatory response triggers a cascade that begins with the activation of TNF and IL-1, alongside other interleukins. This activation proceeds through signal transduction pathways involving NF-κB and Forkhead box O4(FoxO4), adversely affecting muscle mass and function (34, 35). Moreover, estrogen suppresses pro-inflammatory cytokines and up-regulates anti-inflammatory factors (36), meanwhile, indirectly regulating inflammatory responses by regulating microRNAs (37). MicroRNAs, as mediators and pathways of inflammation, may play an essential epigenetic role in the vicious cycle of osteoporosis and vascular calcification (38). However, whether it also plays a significant role in the inflammatory mechanism of T2DM-related sarcopenia deserves further study.

### 2.4 Advanced glycation end-products (AGEs)

Irreversible AGEs are extensively deposited throughout the body, particularly in cartilage, muscular tissues, nerves, and the circulatory system (39, 40). In T2DM, AGE accumulation affects skeletal muscle growth and metabolism through mitochondrial dysfunction and induces cell death (41). Moreover, AGEs can activate intracellular and extracellular signaling cascades, amplifying oxidative stress and inflammatory responses (42). Thus, these effects alter intracellular and extracellular charge distribution, induce protein crosslink formation, and impair muscle contractility (42).

### 2.5 Intramuscular adipose tissue (IMAT)

Heterotopic fat deposition occurs in many insulin target tissues in diabetes patients, including skeletal muscle (43). Firstly, the accumulation of ectopic lipids in skeletal muscle increases the risk of skeletal muscle insulin resistance (44). Secondly, IMAT increases the secretion of pro-inflammatory cytokines, acute-phase proteins, and biologically active lipids such as diacylglycerol and ceramide. This secretion exacerbates the low-grade inflammatory state, damages the insulin signaling pathway, and leads to skeletal muscle mitochondrial dysfunction (44–46). Additionally, mitochondrial abnormalities can cause muscle degeneration and increased ROS production (47). Thirdly, IMAT can activate transcription factors such as NF-κB, exacerbating the breakdown of muscle proteins through subsequent signaling cascades (48). Lastly, as a non-contractile tissue, excessive accumulation of IMAT can affect the elasticity and function of skeletal muscles (49).

### 2.6 Gut microbiota

In patients with T2DM, excess glucose in the intestinal tract leads to a significant reduction in the imbalance of lactobacillus, clostridium, and bifidobacteria populations associated with insulin resistance (50). The imbalance of gut microbiota increases intestinal permeability (51), thereby promoting the entry of microbial metabolites, such as endotoxins (e.g., indophenol sulfate), into the bloodstream. This further encourages the transmission of inflammatory signals and skeletal muscle changes, accelerating muscle aging (52). Moreover, gut microbiota disruption raises intestinal tryptophan metabolite levels, which may increase the incidence of age-related sarcopenia by inducing inflammatory responses in the gut, nervous system, and muscle tissue (53). Healthy gut microbiota-derived phenolic conjugates upregulate the GLUT4 and PI3K pathways, enhancing glucose uptake by muscle fibres and their synthetic metabolic response, thereby increasing muscle mass and reducing skeletal muscle atrophy incidence (54). In addition, gut microbiota transplantation and rational regulation improve microbial diversity, glycaemic control, insulin sensitivity, skeletal muscle quality, and function, showing potential for sarcopenia treatment (50–52).

## 3 Clinical features of sarcopenia in T2DM under related factors

T2DM and sarcopenia are common in the population and often coexist, with possible common factors including age, gender, body mass index, duration of diabetes, blood vessels and nerves, nutritional status and lifestyle. These common factors simultaneously influence these two diseases and exhibit different clinical features.

### 3.1 Age

Those with both T2DM and sarcopenia tend to have a higher mean age than those without sarcopenia (55). Notably, among elderly T2DM patients over 80, nearly 40% have been identified as suffering from sarcopenia (56). Muscle mass declines with age and is negatively correlated with prediabetes prevalence (8), so older people with sarcopenia are more likely to develop diabetes.

### 3.2 Gender

The influence of gender on the occurrence of sarcopenia in individuals with T2DM exhibits variability. Several investigations reported a marked increase in sarcopenia among either males (57–59) or females (60, 61). However, some studies have found that there is no significant gender difference in the incidence of T2DM-associated sarcopenia (62–73). Although there are differences in the research results on the prevalence of sarcopenia in patients with T2DM, most literature shows that the prevalence of sarcopenia in women is lower than that in men (6, 74). In addition to the role of rapid loss of muscle in males due to the decrease in testosterone (75), estrogen plays a vital role in the “anti-degradation” effect of muscle proteins (76). Importantly, it highlights the need for a nuanced understanding of gender-specific factors that may contribute to the prevalence of sarcopenia in diabetic populations, especially the role of female estrogen in this regard.

### 3.3 Body mass index (BMI)

In individuals with T2DM, there is an inverse relationship between BMI and the incidence of sarcopenia (41, 63, 67, 73, 77). Patients with a high body fat percentage but a low BMI were more likely to suffer from muscle atrophy (64). Compared to Caucasians, Asians have a relatively lower BMI and a lower incidence of sarcopenic obesity (78, 79). BMI is positively correlated with muscle PDFF (80). Body composition is a vital assessment factor for sarcopenia in the context of T2DM. Therefore, it is suggested that patients with diabetes should dynamically manage their BMI and body fat rate to prevent sarcopenia.

### 3.4 Diabetes duration

The relationship between the duration of diabetes and sarcopenia is controversial. Some studies show a positive correlation between sarcopenia prevalence and diabetes duration (64, 71, 81), suggesting that it may promote sarcopenia development. However, other studies find no significant link between diabetes duration and sarcopenia (69, 72, 82). Although there are inconsistent findings on this correlation, a double-blind randomized controlled trial observed that T2DM patients receiving metformin treatment walked faster (83). A cross-sectional study also observed that the proportion of sarcopenia in T2DM patients receiving metformin treatment was lower (65). Notably, this mechanism may include indirectly activating AMP-activated protein kinase (AMPK) (84), reducing hyperglycemia and insulin resistance (85), enhancing mitochondrial biogenesis, reducing reactive oxygen species (ROS), and improving muscle fiber atrophy and fibrosis (86).

### 3.5 Blood vessels and nerves

Sarcopenia is closely linked to diabetic peripheral nerve and vascular complications. Research indicates that diabetic sarcopenia accelerates proliferative retinopathy progression (58) and coronary heart disease, stroke, and peripheral arterial disease(PAD) risk (68, 87). Though no significant correlation exists between sarcopenia and microvascular-associated nephropathy incidence in type 2 diabetes patients (69, 88), a retrospective observational study observed that it worsens albuminuria in those with nephropathy (66). Diabetic polyneuropathy can lead to pain and sensory abnormalities, reduce muscle strength (89), and cause muscle atrophy in the affected innervated area. Female patients are more affected than male patients (68). The combination of diabetes and sarcopenia raises the risk of diabetic foot ulcers, which are more severe, have higher Wagner grades, and are more likely to need amputation (90).

### 3.6 Nutritional status and lifestyle

T2DM patients with poor nutritional status are more likely to develop sarcopenia (70, 81, 91). Patients with diabetes and sarcopenia have reduced energy intake (81) and physical activity (57, 65, 92). However, there is no significant difference between developing sarcopenia in terms of smoking and drinking (65, 92). Additionally, a cross-sectional study observed that patients can reduce the incidence of diabetes-related sarcopenia by maintaining adequate physical activity (57). Furthermore, T2DM patients with high vitamin D levels have a protective effect on sarcopenia (82).

## 4 Imaging manifestations

Multiple imaging techniques can be used to assist in the diagnosis of sarcopenia, including DXA, ultrasound, CT, and MRI. Each of these methods has distinct advantages and limitations (Table 1). We will elaborate on the application of these imaging methods from the perspective of the comorbidity of T2DM and sarcopenia.

**Table 1:** Imaging techniques available to detect sarcopenia.

Imaging	Advantages	Limitations
DXA	▪ Inexpensive ▪ Fast imaging speed ▪ low radiation dose	▪ Relatively inaccurate results ▪ Inconsistent data from different DXA devices ▪ 2Dimages ▪ Inability to measure intramuscular fat content
CT	▪ Quantification of fat and muscle tissue ▪ Can be used on specific muscles or the whole body ▪ Can be evaluated on pre-existing CT images	▪ Expensive ▪ Radiation exposure ▪ Lack of normal reference ranges and diagnostic thresholds ▪ Not suitable for large population screening
MRI	▪ Ability to accurately determine muscle quantity and quality ▪ No radiation exposure ▪ Can be used on specific muscles or the whole body	▪ Expensive ▪ Long inspection time ▪ Lack of normal reference ranges and diagnostic thresholds ▪ Applications are limited by multiple factors
US	▪ Portable, fast, few contraindications ▪ no radiation	▪ Results are influenced by body position and muscle status, operator, etc. ▪ Standardization is not uniform

Additional presentation of advantages and limitations. DXA, Dual-Energy X-Ray Absorptiometry; CT, Computed Tomography; MRI, Magnetic Resonance Imaging; US, Ultrasound.

### 4.1 DXA

The imaging principle of DXA is based on the differential absorption of high-energy and low-energy X-rays by different tissues (93). DXA scanning helps evaluate the fat and muscle tissue, as well as bone mineral content, of the entire body or target area (94). Due to its small radiation exposure dose, DXA has a relatively wide application (95).

DXA-derived parameters, such as appendiceal lean mass (ALM) and appendiceal lean mass index (ALMI, calculated as ALM divided by height squared), serve as a pivotal indicator of sarcopenia (96). The Asian Working Group for Sarcopenia (AWGS) has proposed ALMI thresholds of less than 5.5 kg/m^2^ for females and 7.0 kg/m^2^ for males to delineate diminished muscle mass (97). In the comparative analysis of skeletal muscle atrophy assessment based on DXA, patients with T2DM are more likely to develop skeletal muscle atrophy (98, 99), with a significantly higher incidence in elderly T2DM patients than in middle-aged ones, particularly in those aged 75 and above (100).

Bredella et al. (101) conducted a comparative analysis between DXA and CT scans. It revealed a tendency for DXA to overestimate thigh muscle mass, particularly in women with severe obesity. Consequently, this overestimation is attributed to the influence of body thickness and hydration status on DXA measurements. Moreover, the accuracy of DXA measurements may be compromised by factors related to equipment and patient positioning. Thus, it is essential to consider these factors when interpreting DXA results to ensure the reliability of body composition assessments and timely medical interventions (102).

### 4.2 CT

CT imaging measures the X-ray attenuation coefficient, which varies with tissue density and thickness. It can determine the density values of individual muscles or muscle groups, in addition to estimating the area and volume of the muscle (103). There is now considerable research on aspects of sarcopenia in T2DM (Table 2).

**Table 2:** Research results on sarcopenia in T2DM.

References	Research size	Inspection apparatus	Research type	Tissue/structure(s)	MRI imaging mode	Statistical analysis method	Results
Han et al. (115)	420	CT	Prospective cohort study	Mid-thigh, umbilicus		SPSS, chi-square test, Stata	Consistent changes in visceral fat and thigh muscle area were associated with a higher risk of T2DM.
Kim et al. (185)	167	CT	Retrospective cohort study	L3 skeletal muscle		SPSS 21.0, two-sample *t*-test, Cox regression	Sarcopenia was negatively correlated with survival in diabetic patients.
Miljkovic et al. (119)	1,515	pQCT	Prospective cohort study	Calf		Analysis of covariance (ANCOVA), logistic regression, Statistical Analysis System (SAS, version 9.1)	Increased intramuscular fat is associated with the onset of T2DM.
Hildebr et al. (186)	16	HR-pQCT	Cross-sectional study	Tibia		IBM SPSS Statistics software	HR-pQCT can estimate muscle parameters and improve their accuracy.
Baum et al. (187)	9	3.0 T MR	Cross-sectional study	Quadriceps muscle fat	6-echo 3D spoiled gradient echo sequence	SPSS (SPSS, Chicago, Ill)	MRI of lipid and water based on chemical shift coding can reflect early changes in muscle strength.
Scheel et al. (188)	12	1.5 T MR	Cross-sectional study	Soleus muscle	DTI	Linear regression analysis, the software Prism	DTI offers a non-invasive method for assessing changes in fiber type composition in the skeletal muscles of patients with sarcopenia.
Shen et al. (106)	328	1.5 T MR	Cross-sectional study	Skeletal muscle and adipose tissue in the abdominal monolayer section	T1-weighted, spin-echo sequence	SPSS, Two-tailed tests, Pitman’s test	A single abdominal cross-sectional image can reflect the volume of skeletal muscle and adipose tissue throughout the entire body.
Marty et al. (149)	40	3.0 T MR	Prospective cohort study	Thighs muscles	T1-mapping	Matlab (MathWorks, Natick, MA, USA), Bland–Altman plots	T1-mapping enabled a quantitative assessment of muscle fat infiltration, facilitating the evaluation of both acute and chronic skeletal muscle changes in sarcopenia.
Melville et al. (189)	27	3.0 T MR	Prospective cohort study	Quadriceps femoris	T1 weighted 3D gradient echo, T2-mapping and MR spectroscopy	variance (ANOVA)	In patients with sarcopenia, the T2 values of muscle fat infiltration are pathologically increased
Malis et al. (164)	7	1.5 T MR	Prospective cohort study	Triceps Surae muscles	Velocity-Encoded Phase Contrast (VE-PC)	ANOVA, linear regression.	The changes in SR-fiber Angle, SR plane, and shear SR, as well as their ability to predict force and force changes, may reflect functional mechanical changes in skeletal muscle associated with sarcopenia.
Andreu Simó-Servat. et al. (190)	47	Muscle US	Prospective cohort study	TMT		T-test, Pearson’s correlation test, Youden index	TMT less than 0.98 cm was 100% predictive of sarcopenia in elderly diabetic patients.
Chen et al. (181)	84	Muscle US	Prospective cohort study	The US-derived thickness, cross-sectional area, and SWE of the RFM		The Social Sciences version 26.0 (IBM) software, T-test	Muscle CSA is smaller in older T2DM patients with sarcopenia than in non-sarcopenic patients.

CT, Computed Tomography; MRI, Magnetic Resonance Imaging; US, Ultrasound; pQCT, Peripheral Quantitative Computed Tomography; HR-pQCT, High-Resolution Peripheral Quantitative Computed Tomography; DTI, Diffusion Tensor Imaging; TMT, Thigh Muscle Thickness; SWE, Shear Wave Elastography; RFM, Rectus Femoris Muscle; CSA, Cross-Sectional Area.

#### 4.2.1 CT in estimating muscle cross-sectional area and volume

Initially, scholars used the cross-sectional area to represent the volume of muscle. Since CT was first used to measure arm skeletal muscle cross-sectional area in 1979 (104), typical anatomical sites, such as the thigh, proximal femur, and trunk, have been used to measure the cross-sectional area of skeletal muscles (105). Shen et al. (106) introduced a technique to calculate total body skeletal muscle and adipose tissue content from a single conventional abdominal CT scan in 2004. This approach (106) selects the third lumbar vertebrae (L3) as the reference plane, outlining a Region of Interest (ROI) to measure muscle cross-sectional area (CSA), and applying specific threshold criteria (−29 to +150 Hounsfield Units) to segment muscle tissue, the cross-sectional area of muscles obtained based on this calculation method correlate well with total body muscle mass (107, 108), this method eliminates the need for additional examinations when evaluating sarcopenia for patients who already require abdominal CT scans. Derstine et al. (109) have demonstrated that although L3 is the ideal location for skeletal muscle measurements, measurements taken at other levels of T10-L5 can also achieve similar results; thus, the diagnosis of sarcopenia can also be made using existing CT images of the chest and pelvis. Lower skeletal muscle index (SMI) values (CSA/height^2^) are more indicative of sarcopenia (110, 111). A study summarized that the most common diagnostic threshold for diagnosing sarcopenia using SMI derived from abdominal wall muscle tissue was 52–55 cm^2^/m^2^ for males and 39–41 cm^2^/m^2^ for females. This standard has clinical significance for the early identification of high-risk populations, assessment of disease progression, and guidance for personalized treatment (112–114). Han *et al.* used CT studies and found that synchronized changes in visceral fat and thigh muscle area are associated with an increased risk of T2DM (115, 116). Perhaps for T2DM, the measurement scope is not limited to the trunk; limb measurements may also be highly beneficial.

With advancements in algorithms, some scholars have begun to evaluate muscles by their actual volume. However, due to the radiation dose, whole-muscle scanning is not commonly used and is mainly reserved for limb muscles. A South Korean team developed an automatic muscle segmentation software based on the UNETR (U-net Transformer) architecture to quantify thigh muscle volume. This method not only improves computational efficiency, but also enhances the accuracy of muscle volume calculation (117). The study’s results show AI’s strong development potential in this field.

#### 4.2.2 CT assessment of muscle density (MD)

Although cross-sectional area and volume can reflect muscle atrophy, they cannot reflect changes in muscle composition. However, MD can reflect these. MD can be reflected by CT values, which are directly related to the IMAT and can reflect muscle mass to a certain extent (118). The IMAT proportion of T2DM patients is higher than that of normal people (119), and IMAT is related to IR (120). Peripheral quantitative computed tomography (pQCT) and high-resolution peripheral quantitative computed tomography (HR-pQCT) techniques can mitigate the impact of unstable CT results on measurements, thereby yielding more accurate results for skeletal muscle CSA and muscle density. A cross-sectional study of animals and humans evaluated the MD and IMAT produced by intramuscular adipose tissue using HR-pQCT and found that it has good predictive value for the progression of myosteatosis or sarcopenia (121).

By utilizing X-ray beams of varying energy levels to differentiate the penetration and absorption differences of human tissues, Dual-energy CT (DECT) can not only distinguish different components such as skeletal muscle, adipose tissue, and connective tissue. Additionally, it can quantitatively analyze fat infiltration (122). Therefore, it can simultaneously measure multiple parameters, such as muscle cross-sectional area, muscle density, and fat content, which reflect the state of muscles from different perspectives. DECT - determined fat fraction (FF) values strongly correlate with those from MR (123). A study has shown that intermuscular and intramuscular fat increase with age and that intermuscular fat contributes to the increased incidence of T2DM (119). Furthermore, higher muscle fat infiltration correlates with increased insulin resistance (124).

### 4.3 MRI

MRI is an imaging method based on differences in pulse signals released by the relaxation phenomenon of hydrogen nuclei in tissue components following external magnetic field radiofrequency pulses (103). Its multiple sequences and parameters enable it to reflect muscle inflammation, muscle fiber structure, muscle fat infiltration, muscle volume, and ultimately changes in muscle function, thereby reflecting the pathological process of sarcopenia to a certain extent. Many studies have achieved satisfactory results using various imaging sequences (Table 2).

#### 4.3.1 MRI manifestations of inflammation in sarcopenia

Inflammation typically appears earlier in sarcopenia and progresses in conjunction with its progression. Inflammation can manifest as fibrosis, fat replacement, abnormally significant enhancement, and muscle atrophy, but edema is the most common manifestation. Inflammatory edema prolongs T1 and T2 relaxation times of muscle tissue, among which T2 imaging technology is the preferred method for evaluating muscle injury (125). Moreover, combining T1 and T2 values can improve the accuracy of identifying the early stages of muscle edema/inflammation (126). Additionally, the apparent diffusion coefficient (ADC) value of inflamed muscles increases compared to that of normal muscles (93). However, the MRI manifestations of inflammation in T2DM patients with sarcopenia require further research.

#### 4.3.2 MRI manifestations of muscle fiber changes in sarcopenia

Sarcopenia can lead to changes in the type and structure of muscle fibers. Diffusion magnetic resonance imaging (DMRI) can study the microstructure of skeletal muscle and quantify it to a certain extent (127). Fractional anisotropy (FA) values can help distinguish and identify different fiber types. DMRI fiber tracking technology can track the main diffusion characteristic vectors within muscle volume, thereby estimating fiber angle, curvature, muscle fiber length, and cross-sectional area (128). The proportion of type I fibers in the muscles of patients with sarcopenia increases (129). A cross-sectional study observed that DMRI can help elucidate the changes in muscles in the early stages of sarcopenia (130).

T1ρ(T1rho) is a spin–lattice relaxation time in a rotating frame that probes slow molecular motion in muscle tissue macromolecules (131, 132). T1ρ mapping reflects myofibrillar properties and is regarded as a novel quantitative index for assessing myocardial diffuse fibrosis (133). Notably, a prospective cross-sectional observational study shows that it may be an alternative non-contrast method for early T2DM myocardial diffuse fibrosis and is more sensitive than natural T1 (134). Its role in diabetes-associated sarcopenia requires further investigation.

#### 4.3.3 MRI in assessing muscle fat infiltration

MRI reflects the severity of muscle fat degeneration through the degree of muscle fat infiltration. Importantly, it is considered the gold standard for non-invasive quantitative tissue fat content (135). Muscle fat infiltration can monitor the disease progression, guide the rational selection of treatment options, and accurately evaluate the treatment effect (136, 137). Using this approach, a study has shown that the reduction of intramuscular lipids (IMCL) contributes to the improvement of IR (138).

Dixon and 1H-magnetic resonance spectroscopy (MRS) is the preferred sequence for measuring fat infiltration. Dixon technology’s imaging principle is based on the different precession frequencies of fat and water particles in a magnetic field (139, 140). The two or three-point Dixon sequence quantifies the proton density fat fraction (PDFF) with high reliability (141, 142). Magnetic resonance spectroscopy (MRS) is used to distinguish small molecular metabolites according to their chemical shift characteristics under the action of an external magnetic field (143). In contrast, 1H-MRS does not require specialized hardware and is considered the gold standard for non-invasive fat infiltration quantification (144, 145). In patients with T2DM, the muscle fat fraction (FF) is higher than in individuals with normal blood glucose levels (146). A cross-sectional observational study showed a positive correlation between decreased thigh muscle strength and muscle fat infiltration in T2DM patients (147).

T1 mapping quantifies the PDFF in terms of the modulation of T1 values by fat pools. In the case of fat replacement, the amount of fat fraction was positively correlated with a decrease in longitudinal T1 relaxation time (148), T1 mapping can also be used to distinguish between healthy and undernourished muscle (149). In contrast, T2 mapping is based on the T2 relaxation time of human tissue (150, 151), which is significantly elevated in the case of muscle fat infiltration (135). Although there are no diagnostic criteria for muscle fat infiltration in the literature, quantitative T2 values may be influenced by edema changes (152). Regardless, they still serve as a reliable quantitative assessment tool. Diffusion tensor imaging (DTI) evolved from DWI principles and is based on differences in the degree of diffusion of water molecules in various directions (135). In the case of fat infiltration, the muscle fibers appear more clearly on DTI. Furthermore, a prospective cross-sectional observational study has demonstrated a high correlation between fractional anisotropy (FA) and Mercuri grading, which is used clinically to assess the degree of muscle fat infiltration (153). Thus, patients with T2DM have higher apparent diffusion coefficient and lower FA values (154).

#### 4.3.4 MRI in estimating muscle volume

Several sites are used to measure muscle cross-sectional area, including the mid-thigh, the L2 to L5 vertebral levels, the superior mesenteric artery level, and the C3 muscle level. Common indicators for assessing muscle atrophy are CSA, total abdominal muscle area (TAMA), total psoas muscle area (TPA), volume, and the thickness of the muscle (155–158). In exceptional circumstances, temporal muscle thickness can be used to evaluate muscle atrophy, which has been shown to correlate with CSA of the psoas major muscle (159). T2DM can also reduce muscle weight, volume, and cross-sectional area (137), as well as plantar tissue and skin thickness (160).

#### 4.3.5 MRI signs reflecting muscle function

Sarcopenia can lead to changes in skeletal muscle function. Magnetic resonance elastography (MRE) is a technique that captures the dynamic biomechanical properties of skeletal muscle throughout phases of contraction and relaxation, enabling a precise quantitative assessment of skeletal muscle tissue biomechanical properties and reflecting skeletal muscle function (161). Moreover, MRE is reliable for quantitatively measuring muscle stiffness (162, 163). Strain rate tensor imaging is another innovative approach that quantifies muscle contractility and elasticity through strain rate (SR) and strain rate fiber angle (SR-fiber angle). Notably, it shows promise for evaluating muscle mechanical changes in sarcopenia (164, 165). Moreover, 31P-containing metabolite concentrations, measured by magnetic resonance spectroscopy (MRS), are strongly correlated with muscle fat infiltration and subsequent decline in muscle strength (166). The signal changes of Blood-Oxygen-Level-Dependent (BOLD) MRI are also closely related to the level of blood oxygenation. On the one hand, BOLDMRI can reflect the status of muscle microcirculation (167), and on the other hand, it can indirectly reflect the functional status of muscle by dynamically monitoring changes in muscle oxygen metabolism under various physiological conditions (168). Therefore, its performance and clinical application in diabetes sarcopenia warrant further study (169).

### 4.4 Ultrasound (US)

US has become a valuable and reliable tool for assessing muscle volume and mass. It is affordable, allows for real-time dynamic imaging of target tissues during the procedure, and is safe, non-invasive, portable, free from ionizing radiation, and offers high-resolution imaging (170, 171).

#### 4.4.1 US evaluation of muscle volume

Muscle thickness, pennation angle, fascicle length, and muscle cross-sectional area are standard parameters for US assessment of muscle volume. A study proposed the formula for estimating muscle volume: MV = 0.3 × MT + 30.5 × LL (172), where MV = muscle volume, MT = muscle thickness and LL = limb length. Gastrocnemius muscle thickness and muscle fascicle length are highly sensitive and highly accurate for negative results in the detection of sarcopenia (173). A cross-sectional observational study has shown that T2DM patients have significant reductions in plantar tissue and intrinsic foot muscle thickness (174), particularly in the extensor digitorum brevis and first and second intermetatarsal muscles (175).

#### 4.4.2 US assessment of muscle fat infiltration

Muscle echo intensity can reflect the degree of fat infiltration degree (176). It is significantly higher in elderly sarcopenia patients than in young people (177). A cross-sectional observational study quantified rotator cuff muscle fat infiltration via US Backscatter Coefficient (BSC) values, which rose with the Goutallier grade (178). Thus, we hypothesize that US also has the potential to evaluate sarcopenia in T2DM.

#### 4.4.3 Shear wave elastography (SWE) for muscle assessment

SWE is a quantitative technique that determines the absolute elasticity of soft tissues. Notably, it evaluates skeletal muscle stiffness and reflects early changes associated with muscle function (179, 180). A cross-sectional observational study has demonstrated that patients with T2DM and sarcopenia have smaller muscle CSA and increased stiffness values; therefore, SWE is beneficial in identifying sarcopenia in patients with T2DM (181).

## 5 Prospectives

Research on imaging methods in patients with diabetes and sarcopenia is currently limited, with most studies featuring small sample sizes. This limitation may affect the universality and reliability of the research findings. Thus, future studies with larger samples are needed to further explore and validate the application value of imaging methods in this patient population.

Different studies employ various diagnostic techniques, standards, and clinical applications for sarcopenia, making it challenging to compare and integrate research findings. Currently, there is a lack of unified methods for detecting and measuring sarcopenia, which hinders effective comparison and comprehensive analysis across different studies. Developing a feasible solution to address or unify these internal differences is necessary.

Artificial intelligence (AI) algorithms and models have achieved initial results in the detection and evaluation of sarcopenia (182–184),. However, they still lack the application of sarcopenia in the context of T2DM. This gap highlights a promising direction for future research. The combined use of AI algorithms and models during imaging examinations is expected to enhance their role in the study of sarcopenia significantly. Radiomics, a vital research trend, involves extracting and quantifying the characteristic manifestations of sarcopenia in imaging to form a robust database and conduct personalized analysis. Thus, this approach may improve and integrate existing imaging diagnostic criteria, enhance the sensitivity of early identification, and strengthen the accuracy of efficacy and prognosis evaluation.

## 6 Conclusion

Sarcopenia, a disease characterized by age-related decline in muscle mass, strength, or endurance, is increasingly prevalent in patients with T2DM and is closely related to their prognosis. Imaging methods, including DXA, CT, MRI, and US, play a crucial role in evaluating muscle mass, microstructural changes, and function. They are of great value for early diagnosis, understanding of disease pathological processes, assessment of disease severity, evaluation of treatment effectiveness, and prognosis. Thus assisting in the development of personalized treatment plans. By fully leveraging the advantages of each imaging examination, clinicians can gain a more comprehensive understanding of the sarcopenia status in patients with T2DM, which is expected to significantly improve the lifespan and quality of life for these patients.
